# The effect of physiotherapy including frequent changes of body position and stimulation to physical activity for infants hospitalised with acute airway infections. Study protocol for a randomised controlled trial

**DOI:** 10.1186/s13063-020-04681-9

**Published:** 2020-09-21

**Authors:** Sonja Andersson-Marforio, Annika Lundkvist Josenby, Eva Ekvall Hansson, Christine Hansen

**Affiliations:** 1 Department of Health Sciences, Lund University, Margaretavägen 1B, S-22240 Lund, Sweden; 2 Children’s Hospital, Skåne University Hospital, S-22185 Lund, Sweden

**Keywords:** Physiotherapy, Chest physiotherapy, Infants, Bronchiolitis, Pneumonia, Treatment, Randomised controlled trial

## Abstract

**Background:**

Every year, many infants are infected with the respiratory syncytial virus (RSV) or other agents and need hospitalisation due to bronchiolitis. The disease causes much suffering and high costs. Thus, it is important that the treatment methods are both effective and cost-efficient. The use of different physiotherapy treatment methods is debated, and not all methods are evaluated scientifically. The clinical praxis in Sweden that includes frequent changes of body position and stimulation to physical activity has not previously been evaluated. The aim of this clinical study is to evaluate this praxis.

**Methods:**

This study is a clinical two-centre individually randomised controlled trial (RCT) with three parallel groups. The participants will be randomly assigned to an individualised physiotherapy intervention, a non-individualised intervention, or a control group. All three groups will receive the standard care at the ward, and the two intervention groups will receive additional treatment, including different movements of the body. The primary outcome measure is a clinical index based on determinants for hospitalisation. Baseline assessments will be compared with the assessments after 24 h. The secondary outcome measures include vital signs, the parents’ observations, time spent at the hospital ward, and referrals to an intensive care unit. We also want see if there is any immediate effect of the first intervention, after 20 min.

**Discussion:**

This study will add knowledge about the effect of two physiotherapy interventions that are commonly in use in Swedish hospitals for infants with bronchiolitis or other acute lower respiratory tract infections.

**Trial registration:**

ClinicalTrials.gov NCT03575091. Registered July 2, 2018—retrospectively registered.

## Background

Infants with acute breathing difficulties due to a lower respiratory tract infection such as bronchiolitis or pneumonia often have obstruction of the smaller airways caused by oedema and excess mucus production [1]. This can lead to increased work of breathing with exhaustion of respiratory muscles, feeding difficulties, and increases the risk of developing respiratory distress. Affected infants may need treatment at an intensive care unit (ICU). The hospital care for the children with bronchiolitis involves a high cost for families and health care organisations around the world [2], as this is a common illness in the winter season [3]. The treatment in hospitals is most often supportive, such as supplementing oxygen and fluids [4].

Sometimes, physiotherapy treatment is used to reduce the symptoms of infants who are hospitalised with bronchiolitis. Physiotherapy methods aim at moving and evacuating mucus from the airways in order to reduce work of breathing, increase gas exchange, and increase lung volumes [5–7]. The aim of physiotherapy treatment has also been to reduce time to clinical stability or reduce the duration of a hospital stay [8, 9]. There is no clear consensus about the efficiency of physiotherapy treatment on the whole for this patient group, and some of the treatment methods are questioned or not recommended at all [10, 11].

Many of the physiotherapeutic interventions that are described in international literature for infants hospitalised with breathing difficulties such as bronchiolitis or pneumonia can be described as passive: the physiotherapist places the child in different drainage or resting positions, applies manual pressure to the child’s chest wall, or uses technical devices like continuous positive airway pressure (CPAP) or positive expiratory pressure (PEP) [5, 8, 9, 12–21]. In Sweden, however, physiotherapy for infants in hospital with acute airway infections most often involves stimulation to physical activity and frequent changes of the body position combined with other methods [22]. This praxis is supported by general physiological principles of the positive effect on lung function of the change of body positions and physical activity [23–25], but these methods have not been evaluated scientifically for this patient group before, and that is why this study was designed.

There are studies that indicate a positive effect of prone position (lying on their stomach) for children with respiratory problems. Most of these studies, however, are made on premature infants who are mechanically ventilated [26, 27]. The present study aims at investigating changes of body position on full-term children who breathe spontaneously.

## Methods/design

### Aim

The present study protocol describes a randomised control trial that aims to compare the effect of an individualised physiotherapy intervention, a non-individualised intervention and a control group receiving standard care, in hospitalised infants 0–24 months of age.

### Study design

This is a clinical two-centre individually randomised controlled trial with three parallel groups.

**Fig. 1:**
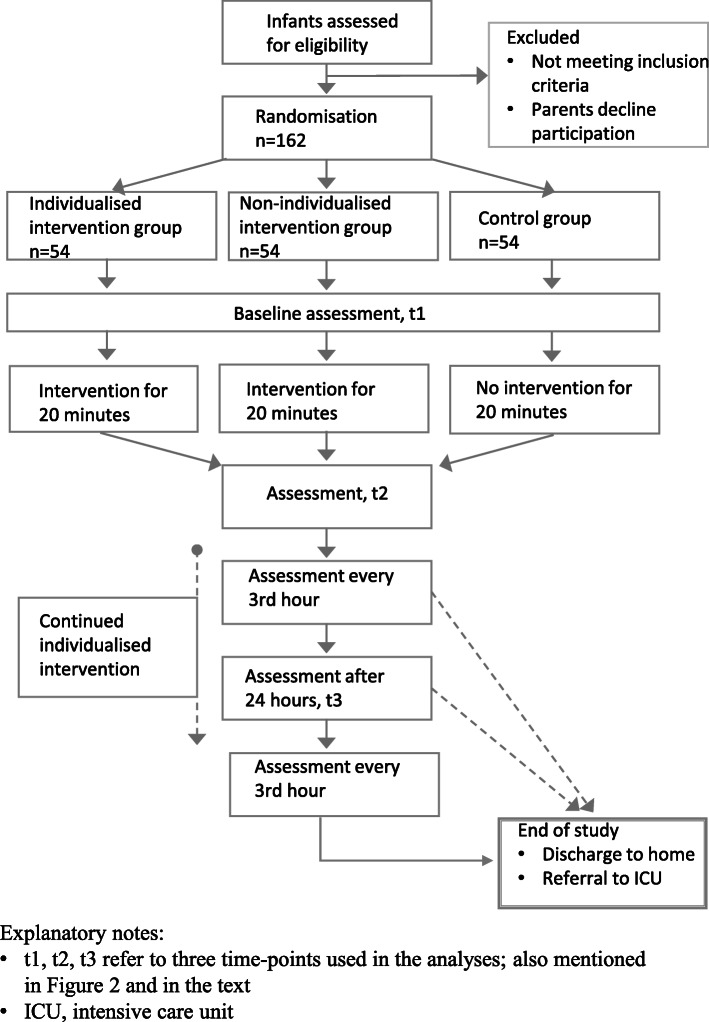
Flow chart of the study design

**Fig. 2:**
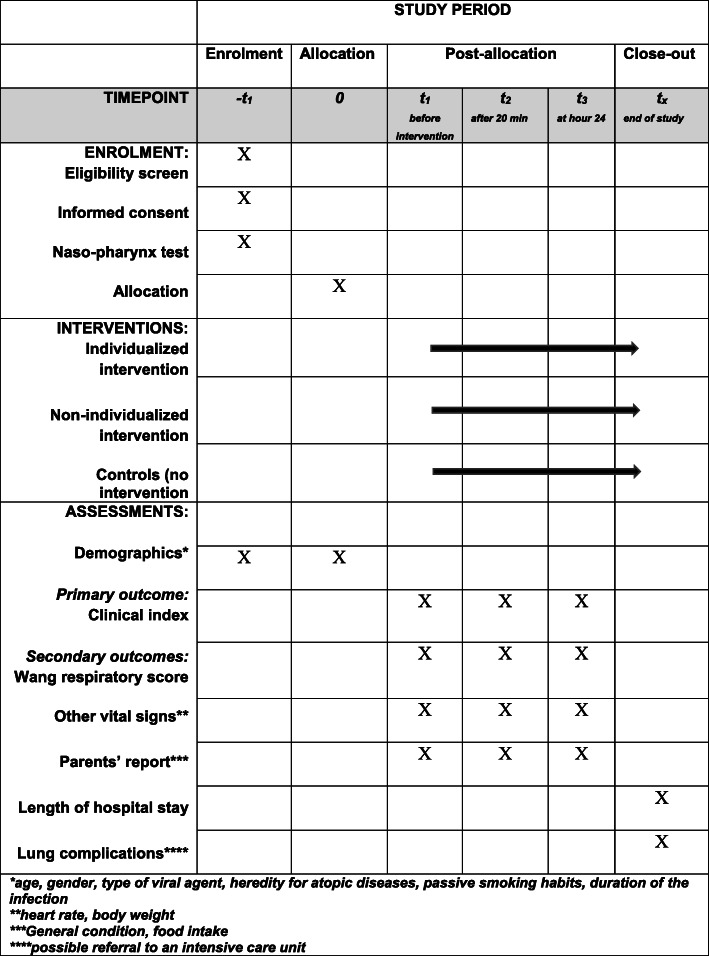
The SPIRIT figure of enrolment, interventions, assessments, and outcomes

We plan to include 162 infants who will be randomised to either the individualised physiotherapy intervention group, the non-individualised intervention group, or the control group, 54 in each group. In the flow chart (Fig. 1) and in the Standard Protocol Items: Recommendations for Interventional Trials (SPIRIT) figure (Fig. 2), the overall study design is described, which is in agreement with the SPIRIT 2013 checklist (see Additional file 1). Any significant changes of the protocol will be registered at ClinicalTrials.gov and communicated to the staff involved through the contact staff on the both sites.

### Setting

The study will take place at the children’s wards in two hospitals in the south of Sweden (Malmö and Växjö). One hospital has a catchment area of about 500,000 inhabitants, the other of about 100,000 inhabitants. The two hospitals are situated about 200 km apart.

### Inclusion of participants

Potential participants will be identified and recruited by the staff in the paediatric wards. Inclusion criteria are as follows: age 0–24 months, hospitalised on the basis of acute airway infection, born in gestational week 35 or later. Patients must be included within 24 h of hospital admission. At least one of the parents/guardians needs to understand written Swedish, English, Arabic, or Persian. Exclusion criteria are as follows: previous respiratory or cardiac diagnoses.

To include adequate participants, the study will be mentioned at two pulse meetings a day in the wards, and all staff members will be trained in the study design. Some staff members will get additional training to support their colleagues. The main investigator will regularly visit the wards in person and will have telephone communication in between the visits to remind the staff and to answer questions.

First, the parents will receive written information about the study (text in Swedish, Arabic, English, or Persian). At that time and when they have had the opportunity to read the information, they may ask questions and get additional information from the staff. In order to admit their child into the study, the parent/s will sign a written consent form. The participation in the study will end when the infant is either discharged to the home or referred to an intensive care unit or if the parents decide to withdraw the participation of their child.

### Randomisation

When a child is included in the study, it will be individually randomised to one of the three groups. A statistician who is independent of the study has performed randomisation in blocks, stratified by the two sites. The statistician prepared sealed paper envelopes, numbered in sequence, that are kept in a locked safe accessible only for the researchers responsible for the study. A small number of envelopes will be brought to the study binders at the two hospital wards when needed. The staff that will include an infant in the study after parental consent is instructed to open the envelope with the lowest number, which will be the top envelope in the binder.

### Outcome measures and assessors

#### Baseline data

Participant characteristics will be collected from (i) interview with parents (heredity for atopic diseases, passive smoking habits, duration of the infection) and (ii) medical record (gender, age, possible viral agent: respiratory syncytial virus or influenza).

To collect the main data, the nursing staff will make a clinical assessment at study start (t1) and the second assessment 20 min later (t2), following directly after the intervention/interval. See also Fig. 1. The same person will perform these two assessments. The following assessments will be performed every three hours for the rest of the infant’s hospital stay. They will be performed by the clinical staff available at that time. In the intervention groups, it will be a different member of staff who will perform the intervention from the one who makes the first two assessments. All members of the staff will be trained on how to perform the assessments, which in many aspects are identical to the standard protocol at the ward. Once daily, the clinical staff will report if the infant has received any inhalations (in which case the medical record is scanned for more information on this) and also if the infant is actively moving about in the room or not. See Additional file 2 for the assessment protocol in Swedish.

#### Primary outcome measures

A clinical index combining levels of oxygen saturation, oxygen concentration (need for oxygen supplementation), high nasal flow treatment, and oral fluid intake (compared to tube feeding) was constructed; see Table 1. It is possible to achieve a total score between 0 (worst condition) and 11. The primary outcome is a composite index that the research group constructed for this study, based on the factors that determine whether the infant needs hospitalisation [28]. The items in the index were chosen because of their clinical implication. They are based on objective values and are therefore not likely to differ between different assessors. The index has not been validated, but was tested in a small pilot study (unpublished material), where the clinically relevant change of two points was determined by clinical reasoning.

**Table 1:** The components of the clinical index used as the primary outcome measure

Outcome	Definition	Measure	Registration time	Score
**Oxygen saturation**	%	By pulse oximetry	At t1, t2, and every subsequent 3rd hour	
	≥ 96			2
	90–95			1
	≤ 89			0
**Oxygen concentration**	%		At t1, t2, and every subsequent 3rd hour	
	21			4
	22–30			3
	31–40			2
	41–50			1
	≥ 51			0
**High nasal flow**	Litres/kg/min		At t1, t2, and every subsequent 3rd hour	
	0			2
	0.1–1			1
	1.1–2			0
**Oral fluid intake**	%^a^	g (weight when breast feeding) or ml (by bottle)	At every feeding session	
	100			3
	51–99			2
	1–50			1
	0			0

^a^Per cent of the calculated daily need

The daily fluid need will be calculated according to nutrition guidelines [29, 30]. The oral fluid intake for analysis at hour 24 will be the collected intake during the first 24 h in the study.

#### Secondary outcome measures

The clinical status of the participants will be assessed by the nursing staff and the parents using the measures in Table 2. Wang score [31] is an observational scale where the nursing staff scores the infants 0–3 where 3 is most severe. The scoring system includes respiratory rate, wheezing, retractions/nasal flaring, and general condition. It has been validated in clinical studies [31–33]. The respiratory rate, retractions, and wheezing is commonly used in studies of bronchiolitis, and the inter-observer agreement is good: 93.1% with a weighted kappa of 0.72 (95% CI 0.66–0.78) [33].

**Table 2:** The secondary outcome measures, reported by the clinical staff unless otherwise stated

Secondary outcome	Measure	Registration time
Wang respiratory score [31]		
Respiratory rate	Manual count, per minute. Scale 0–3 where 3 is worst	At baseline, 20 min later and every 3rd hour
Wheezing sound	Clinical observation. Scale 0–3 where 3 is worst	At baseline, 20 min later and every 3rd hour
Retractions/nasal flaring	Clinical observation. Scale 0–3 where 3 is worst	At baseline, 20 min later and every 3rd hour
General condition	Clinical observation. Scale 0–3 where 3 is worst	At baseline, 20 min later and every 3rd hour
General condition (parents’ report)	Observation. Scale 0–10 where 10 is worst	At baseline, 20 min later and every 3rd hour during daytime
Food intake (parents’ report)	Observation. Scale 0–2 where 2 is worst	At baseline, 20 min later and every 3rd hour during daytime
Body weight	Naked weight on scales, g	Once daily
Heart rate	Counts per minute, by pulse-oximetry, probe on the foot	At baseline, 20 min later and every 3rd hour
Time to recovery	Time at hospital, hours	At the end of the study
Lung complications	Referral to intensive care unit—yes/no	At the end of the study

The infant’s food intake will be reported by the parents by a custom-made three-level scale 0–2 where 0 = no self-contained eating at all, 1 = eats less than usual, and 2 = eats as usual. The infant’s general condition will be reported by the parents using a visual numeric rating scale 0–10 where 0 = as usual and 10 = very affected/ill. The numeric rating scale 0–10 has been validated for assessing pain [34], for cancer-related symptoms such as pain and severe tiredness [35], and for postoperative nausea [36] and patient-specific function [37]. It has also been used to assess different symptoms in studies although not validated for this, like well-being, depression, fatigue, and anxiety [38].

Body weight will be used to calculate the daily food/fluid need, and heart rate might show cardiorespiratory load or stress.

Time at hospital and referrals to ICU as a measure of severity of the disease have also been used in other studies [5].

#### Interventions

The position for all infants at baseline is supine on the bed, that is, lying on their backs. Directly following the baseline assessment, there will be 20 min of interventions for the two intervention groups and 20 min of no extra intervention for the controls. All participants will receive the standard care at the ward without limitation, and the participants in the intervention groups will have physiotherapy treatment in addition to the standard care.

*The standard care* at the wards is information to the parents about the importance of fluid intake for their infant, oxygen supplementation, nose drops and suctioning, high nasal flow, inhalations, fluid supplementation, and analgesics, according to need.

The different interventions were chosen based on what is routinely carried out by physiotherapists or nursing staff in hospitals in Sweden [22], including an individualised physiotherapy treatment and a reduced non-individualised treatment. We expect that the individual intervention might be more efficient, but at the same time want to know whether the non-individualised treatment would be sufficient for these patients, indicated by outcomes in the non-individualised group showing a both statistically and clinically relevant improvement compared to the control arm.

In the individualised intervention group, the intervention is minimally given once daily by the physiotherapist, and in the non-individualised intervention group, the intervention is minimally given by the staff once. The randomised treatment, or indeed what will be performed in the control group, will not be monitored, so there might be differences in adherence to the interventions between parents. Our intention, however, is to evaluate the current practice that includes instructions and encouragements to the parents. This attitude is in accordance with a so-called pragmatic RCT, as our intention is to apply the results to the usual care setting [39].

#### Controls

In the control group, the infants will receive no additional body movements, and the parents will not be given extra encouragement to lift up their child.

#### The individualised intervention

**Fig. 3:**
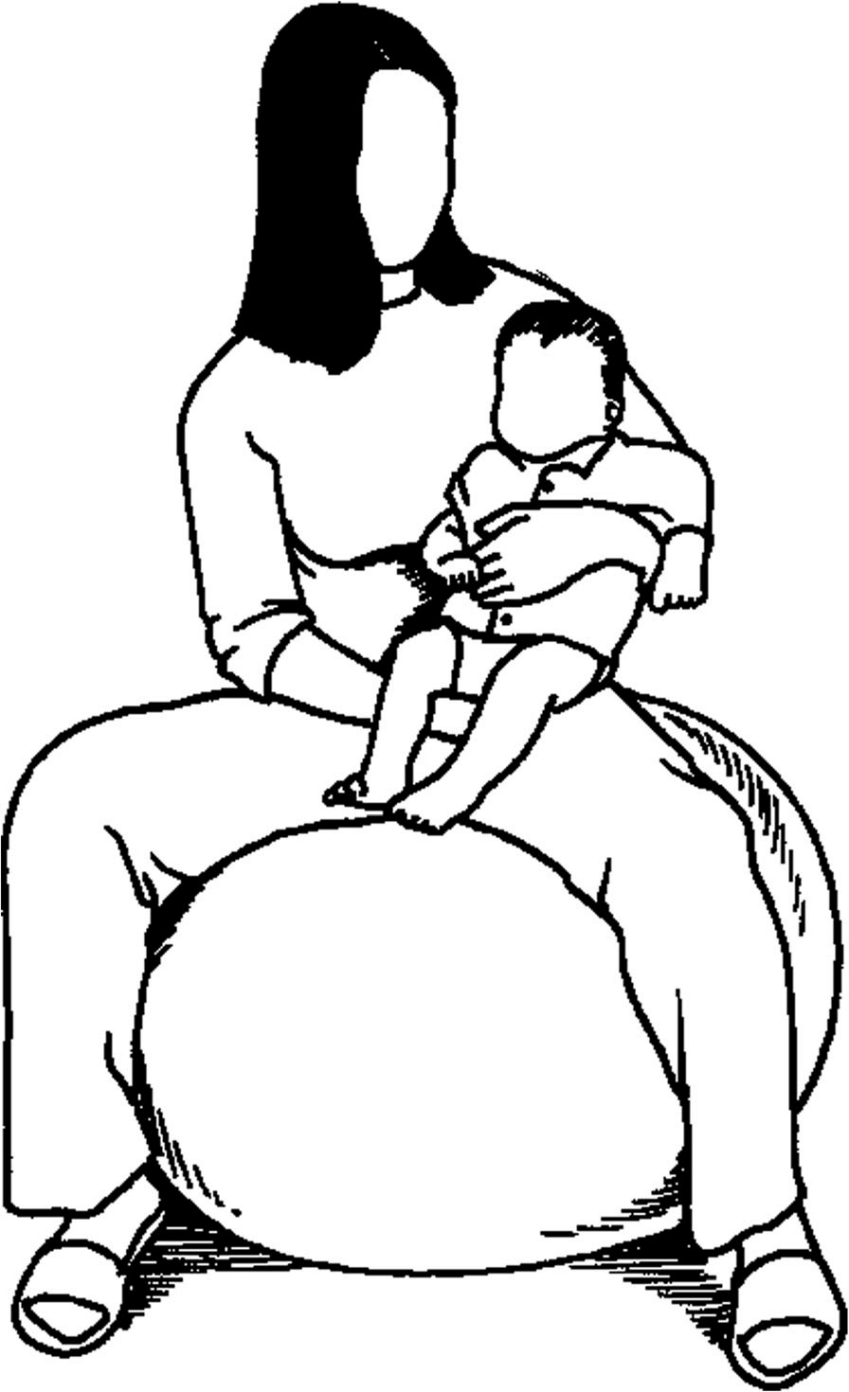
Example of movement in the individualised intervention group. The physiotherapist bounces on a large ball while holding the infant in different body positions approximately 20 s in each position

The individualised physiotherapy intervention consists of a standardised and individualised programme for 20 min. The individualised intervention will be performed by the physiotherapist once daily, or more often according to his or her judgement. The physiotherapist will lift up the infant in their arms and place the infant in different positions in their arms while bouncing on a large ball; see Fig. 3. The physiotherapist will also stimulate to physical activity by placing the infant in a prone position (lying on their stomach), stimulate the infant to actively move their arms or legs, move the infant’s arms or legs, and supply thoracic compressions, and they may give inhalations and manual cough support on the infant’s belly and chest. The physiotherapist may also suggest other treatments, such as different inhalations or CPAP. They will also teach the parents how to continue this procedure by themselves and will encourage them to do the programme every other hour during the infant’s waking time. The written information to the parents is supplemented in Additional file 3.

#### The non-individualised intervention

**Fig. 4:**
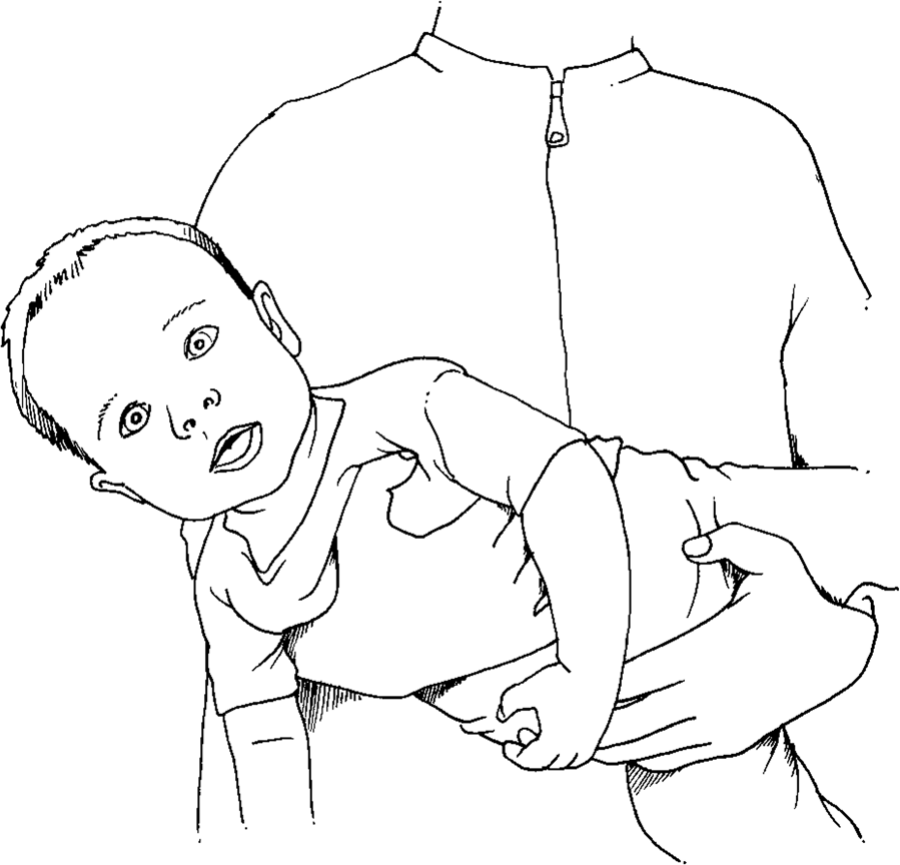
Example of body position in the non-individualised intervention group, where the nursing staff changes the infant’s body position in their arms

The non-individualised intervention can be described as a reduced version of the individualised intervention, without using the large ball, and inhalations given typically only in the upright position, not in frequently changing positions in the arms. The intensity of the treatment will also typically be reduced, as the staff will only have to perform the intervention once, but can choose to repeat it. One member of the clinical staff responsible for the patient at the ward will perform a standardised programme that includes lifting up the infant in their arms to change the body position of the infant, moving the arms and legs of the infant, and giving manual cough support on the belly and chest; see Fig. 4. They will teach the parents to continue to regularly change the infant’s body position themselves. The written information to the parents is supplemented in Additional file 4. The intervention will be performed once at the beginning of the study. The nursing staff can opt to repeat the intervention more times or encourage the parents as often they wish or not at all. All nursing staff at the ward will be trained in the programme. The non-individualised intervention is based on what is commonly performed by the nursing-staff in Swedish hospitals, as instructed by physiotherapists.

#### Statistical and sample size calculations

Descriptive statistics will be shown as mean with standard deviation or median with minimum and maximum values for continuous and ordinal variables where appropriate. Number with percent will be shown for categorical variables.

The primary outcome is clinical index at 24 h. The time is chosen since our experience is that most infants will remain at hospital at that time and an effect should have been shown. The effect of the individualised physiotherapy intervention, the non-individualised intervention, and a control group receiving standard care on clinical index at 24 h will be assessed by an ANCOVA model with adjustment for baseline clinical index, and post hoc test for the different group combinations will be performed.

The secondary outcomes, including clinical index at 20 min, will first be assessed by an overall test and if the overall test is shown significant post-hoc tests will be performed. A *p* value below 0.05 will be considered significant for the overall test. Continuous and ordinal variables will be assessed by ANCOVA model with adjustment for baseline values where possible, ANOVA, or Kruskal-Wallis test, with *t* test or Mann-Whitney *U* test as post hoc tests, where appropriate. Categorical variables will be assessed by Fisher’s exact test. Explorative subgroup analyses by gender will be performed. To increase the knowledge about what time the infants will recover, data are collected every 3 h. We will analyse the time the infants recover by means of Fisher’ exact test and Kaplan-Meier analysis. IBM SPSS Statistics 24, or higher, Windows (IBM Corporation, Armonk, NY, USA) will be used to perform the statistical analyses.

The primary analysis will include all participants with available outcome data at 24 h. Drop-outs will not be included in this analysis. A dataset with the imputed values might also be analysed. Simple imputation, mean of the value before and after the missing value or last value carried forward from the assessment three hours earlier, will be performed if data is missing at hour 24 and the patient is still at hospital. If an infant will be discharged from the ward before hour 24, that participant will be excluded from this analysis and will be analysed in the same way as the other potential drop-outs.

The sample size is calculated on a two-sided two sample *t* test with equal variance. The mean difference that we want to be able to detect in clinical index at 24 h is 2. A previous pilot study (unpublished material) has shown that the standard deviation is approximately 2.8. The power probability for the test is 0.80, and to be able to correct for the three primary tests, comparing the individualised physiotherapy intervention, the non-individualised intervention, and a control group receiving standard care with each other, the type I error probability in the calculation is 0.016, confidence interval 0.95. To be able to detect the difference, 43 evaluable patients in each group is needed. The calculation is performed in SAS Enterprise Guide 6.1 for Windows (SAS Institute Inc., Cary, NC, USA). Incomplete observations and drop-outs are expected to amount to about 20%. Thus, a total of 162 patients are needed to be included in the study. The drop-outs will be analysed according to age, possible viral infection, gender, allocation group, and severity of illness at admission to the ward (saturation, respiratory rate and heart rate or more data if collected). We plan to perform an interim analysis of the data obtained after inclusion of 50% of the estimated sample size in order to check the assumptions about the standard deviation and recalculate the sample size. In that analysis, we will also be able to address possible safety concerns in the study, although we do not anticipate any harmful outcome. That analysis will be performed by a statistician independent of the study, and the result will only be presented to the research group at this stage if any harmful outcome will be detected in the intervention groups, such as significant inferior outcomes in the intervention groups compared to the control arm. Further instructions will be discussed by the trial team before the interim analysis.

#### Data management

Data from all assessments will be decoded and stored in an external hard drive that is kept in a safe to which only the researchers responsible have access. The hard drive has no connection to the Internet. The data from the paper protocols will be manually entered onto the database by the primary investigator (SAM). The accuracy of the data in randomly chosen protocols will be double-checked by two other researchers in the group. During the period of entering data, the hard drive will be regularly backed-up to a USB memory, which will also be kept in a safe. All files will be saved for at least 10 years after completion of the study. The data will be disseminated by publication in scientific journals.

## Discussion

Infants hospitalised due to acute respiratory failure caused by bronchiolitis and pneumonia is a recurring group of patients in paediatric departments. Hospitalisation is a burden for both the families and for society, and possible treatments that reduce the need for hospitalisation by reducing the work of breathing and increase the general condition of the infant are called for, as well as finding treatments that alleviate the current situation for these infants during hospitalisation.

Our study focuses on a possible effect of an individualised physiotherapy intervention and a non-individualised intervention compared to the standard care (without additional movements).

We chose to include infants without previously diagnosed respiratory and cardiac diseases, in order to evaluate the effect of physiotherapy in a homogenous group of patients, in a real-life setting in two departments.

We chose the individualised physiotherapy intervention because that is the most common physiotherapy treatment method in Sweden [22] and previously not evaluated. The non-individualised physiotherapy intervention can be used by the nursing staff and is less time-consuming and often used, while the control group will receive treatment as recommended in earlier studies [4, 40].

Blinding a physiotherapy study is difficult. In some studies, this problem was solved by bringing all infants to a closed room to do the physiotherapy intervention or nothing, without the parents and nursing staff knowing what was done [5, 8, 19]. In our study, we wanted to evaluate the commonly used methods that often involve parents’ actions, and blinding of parents to participants, care providers, or assessors was not possible due to the nature of the intervention. To minimise possible bias, one person will perform the intervention and another person will evaluate the result of the different interventions (make the assessment). The statistician involved in the analyses will be blinded.

Block randomisation was chosen to ensure equal numbers of participants in the three groups over time as this can compensate for possible differences in treatment in the two sites, but even for possible changes in the surroundings over time (learning curve, change of staff or temporary severity of the infection). A drawback with equal numbers in the three groups is that exclusions from the physiotherapy intervention group is more likely to be made than from the other groups due to absence of a physiotherapist on occasions, for example at nights and absence due to leave. We have, however, calculated generously on the drop-out rate to compensate for this, and the drop-outs will be closely analysed as described earlier.

There is a need for an easy and clinically relevant scoring system for assessing infants with breathing difficulties in hospitals. Our primary outcome is a clinical index that the research group constructed, based on the factors that determine whether the infant needs hospitalisation [28]. The items in the clinical index were chosen because of their clinical implication. They are based on objective values and are therefore not likely to differ between different assessors. Similar outcome measures were also used by Rochat et al. [9].

For secondary outcomes, we included the Wang score, since it is often used in other studies of infants with acute respiratory infections [5, 8, 19] and has been validated. Furthermore, the parents’ report on the infants’ well-being and eating ability was included. The parents have unique knowledge of their own child, which we thought could make a valuable addition to the nursing staff’s assessment. The parents’ report has not, to our knowledge, been used before in studies of infants with breathing difficulties in hospitals. These two scores were constructed by the research group for this purpose and are not validated for this use. The numeric rating scale 0–10 has been used before to assess different symptoms although not validated for this, like well-being, depression, fatigue, and anxiety [38], and by using it in this study, we hope to gain at least some information about the parents’ assessment of their infant’s general well-being. The outcome measures were chosen with the aim to detect many different types of change in the infants’ status.

The result from this RCT will add new knowledge about the effect of physiotherapy interventions on infants hospitalised with acute breathing difficulties due to respiratory infections. It may also contribute to an increased understanding of how to perform studies on infants in a clinical setting in hospitals.

## Trial status

The recruitment of participants started on 1 November 2017 and the trial is expected to continue until May 2021.

On submission for publication, version 1.0 of the protocol was being used. April 2020.

## Supplementary information

**Additional file 1.**

**Additional file 2.**

**Additional file 3.**

**Additional file 4.**

**Additional file 5.**

## Data Availability

The datasets analysed during the current study are available from the corresponding author on reasonable request.

## Abbreviations

RSV: Respiratory syncytial virus
RCT: Randomised control trial
ICU: Intensive care unit
CPAP: Continuous positive airway pressure
PEP: Positive expiratory pressure

## References

[1] Midulla F, Nicolai A, Moretti C, Eber E, Midulla F (2013). Acute viral bronchiolitis. ERS Handbook of paediatric respiratory medicine. 1 ed.

[2] Nair H, Simoes EA, Rudan I, Gessner BD, Azziz-Baumgartner E, Zhang JS (2013). Global and regional burden of hospital admissions for severe acute lower respiratory infections in young children in 2010: a systematic analysis. Lancet (London, England).

[3] Munoz-Quiles C, Lopez-Lacort M, Ubeda-Sansano I, Aleman-Sanchez S, Perez-Vilar S, Puig-Barbera J (2016). Population-based analysis of bronchiolitis epidemiology in Valencia, Spain. Pediatr Infect Dis J.

[4] Florin TA, Plint AC, Zorc JJ (2017). Viral bronchiolitis. Lancet.

[5] Gajdos V, Katsahian S, Beydon N, Abadie V, de Pontual L, Larrar S (2010). Effectiveness of chest physiotherapy in infants hospitalized with acute bronchiolitis: a multicenter, randomized, controlled trial. PLoS Med.

[6] Lannefors L (2010). Cystic fibrosis-long term results of a treatment package including preventive physical exercise [dissertation].

[7] Postiaux G, Zwaenepoel B, Louis J (2013). Chest physical therapy in acute viral bronchiolitis: an updated review. Respir Care.

[8] Gomes EL, Postiaux G, Medeiros DR, Monteiro KK, Sampaio LM, Costa D (2012). Chest physical therapy is effective in reducing the clinical score in bronchiolitis: randomized controlled trial. Rev Bras Fisioter.

[9] Rochat I, Leis P, Bouchardy M, Oberli C, Sourial H, Friedli-Burri M (2012). Chest physiotherapy using passive expiratory techniques does not reduce bronchiolitis severity: a randomised controlled trial. Eur J Pediatr.

[10] Gomes GR, Donadio MF. Effects of the use of respiratory physiotherapy in children admitted with acute viral bronchiolitis. Archives de Pédiatrie. 2018;25(6):394–98. 10.1016/j.arcped.2018.06.004 30064712

[11] Roque i Figuls M, Gine-Garriga M, Granados Rugeles C, Perrotta C, Vilaro J (2016). Chest physiotherapy for acute bronchiolitis in paediatric patients between 0 and 24 months old. Cochrane Database Syst Rev..

[12] DiDario AG, Whelan MA, Hwan WH, Yousef E, Cox TJ, Oldham HM (2009). Efficacy of chest physiotherapy in pediatric patients with acute asthma exacerbations. Pediatric Asthma Allergy Immunol.

[13] Giannantonio C, Papacci P, Ciarniello R, Tesfagabir MG, Purcaro V, Cota F (2010). Chest physiotherapy in preterm infants with lung diseases. Ital J Pediatr.

[14] Jacinto CP, Gastaldi AC, Aguiar DY, Maida KD, Souza HC (2013). Physical therapy for airway clearance improves cardiac autonomic modulation in children with acute bronchiolitis. Brazilian J Phys Ther.

[15] Kugelman A, Feferkorn I, Riskin A, Chistyakov I, Kaufman B, Bader D (2007). Nasal intermittent mandatory ventilation versus nasal continuous positive airway pressure for respiratory distress syndrome: a randomized, controlled, prospective study. J Pediatr.

[16] Lukrafka JL, Fuchs SC, Fischer GB, Flores JA, Fachel JM, Castro-Rodriguez JA (2012). Chest physiotherapy in paediatric patients hospitalised with community-acquired pneumonia: a randomised clinical trial. Arch Dis Child.

[17] Milesi C, Matecki S, Jaber S, Mura T, Jacquot A, Pidoux O (2013). 6 cmH2O continuous positive airway pressure versus conventional oxygen therapy in severe viral bronchiolitis: a randomized trial. Pediatr Pulmonol.

[18] Paludo C, Zhang L, Lincho CS, Lemos DV, Real GG, Bergamin JA (2008). Chest physical therapy for children hospitalised with acute pneumonia: a randomised controlled trial. Thorax..

[19] Postiaux G, Louis J, Labasse HC, Gerroldt J, Kotik AC, Lemuhot A (2011). Evaluation of an alternative chest physiotherapy method in infants with respiratory syncytial virus bronchiolitis. Respir Care.

[20] Pupin MK, Riccetto AG, Ribeiro JD, Baracat EC (2009). Comparison of the effects that two different respiratory physical therapy techniques have on cardiorespiratory parameters in infants with acute viral bronchiolitis. J Bras Pneumol.

[21] Thia LP, McKenzie SA, Blyth TP, Minasian CC, Kozlowska WJ, Carr SB (2008). Randomised controlled trial of nasal continuous positive airways pressure (CPAP) in bronchiolitis. Arch Dis Child.

[22] Andersson-Marforio S, Hansen C, Ekvall Hansson E, Lundkvist Josenby A. A survey of the physiotherapy treatment methods for infants hospitalised with acute airway infections in Sweden. Eur J Phys. 2019. 10.1080/21679169.2019.1663925.

[23] Dean E, Frownfelter DL, Dean E (2013). Body positioning. Cardiovascular and pulmonary physical therapy: evidence to practice.

[24] Dean E, Butchers S, Frownfelter D, Dean E (2013). Mobilization and exercise: physiological basis for assessment, evaluation and training. Cardiovascular and pulmonary physical therapy: evidence to practice.

[25] Lumb AB (2017). Nunn’s applied respiratory physiology.

[26] Gillies D, Wells D, Bhandari AP (2012). Positioning for acute respiratory distress in hospitalised infants and children. Cochrane Database Syst Rev.

[27] Baudin F, Emeriaud G, Essouri S, Beck J, Portefaix A, Javouhey E (2019). Physiological effect of prone position in children with severe bronchiolitis: a randomized cross-over study (BRONCHIO-DV). J Pediatr.

[28] The National Institute for Health and Care Excellence (NICE). Bronchiolitis in children: diagnosis and management. NICE guideline NG9 [Internet]. Manchester: The National Institute for Health and Care Excellence; 2015 [cited 2019 16 August]. Available from: https://www.nice.org.uk/guidance/ng9/chapter/1-Recommendations#when-to-admit.

[29] Läkemedelskommittén i Västra Götalandsregionen. Vätske- och nutritionsbehandling [Internet]. [Therapy group Fluids and nutrition] Göteborg: Läkemedelskommittén i Västa Götalandsregionen; 2011 [cited 2019 30 Oktober]. Available from: https://www.vgregion.se/halsa-och-vard/vardgivarwebben/vardriktlinjer/lakemedel/terapirad/vatskor-och-nutrition/.

[30] Shaw V, Lawson M, Shaw V, Lawson M (2007). Nutritional assessment, dietary requirements, feed supplementation. Clinical paediatric dietetics.

[31] Wang EE, Milner RA, Navas L, Maj H (1992). Observer agreement for respiratory signs and oximetry in infants hospitalized with lower respiratory infections. Am Rev Respir Dis.

[32] Chin HJ, Seng QB (2004). Reliability and validity of the respiratory score in the assessment of acute bronchiolitis. Malaysian J Med Sciences : MJMS.

[33] Gajdos V, Beydon N, Bommenel L, Pellegrino B, de Pontual L, Bailleux S (2009). Inter-observer agreement between physicians, nurses, and respiratory therapists for respiratory clinical evaluation in bronchiolitis. Pediatr Pulmonol.

[34] Hawker GA, Mian S, Kendzerska T, French M (2011). Measures of adult pain: Visual Analog Scale for Pain (VAS Pain), Numeric Rating Scale for Pain (NRS Pain), McGill Pain Questionnaire (MPQ), Short-Form McGill Pain Questionnaire (SF-MPQ), Chronic Pain Grade Scale (CPGS), Short Form-36 Bodily Pain Scale (SF-36 BPS), and Measure of Intermittent and Constant Osteoarthritis Pain (ICOAP). Arthritis Care Res (Hoboken).

[35] Oldenmenger WH, de Raaf PJ, de Klerk C, van der Rijt CC (2013). Cut points on 0-10 numeric rating scales for symptoms included in the Edmonton Symptom Assessment Scale in cancer patients: a systematic review. J Pain Symptom Manag.

[36] Wikström L, Nilsson M, Broström A, Eriksson K (2019). Patients’ self-reported nausea: Validation of the Numerical Rating Scale and of a daily summary of repeated Numerical Rating Scale scores. J Clin Nurs.

[37] Horn KK, Jennings S, Richardson G, Vliet DV, Hefford C, Abbott JH (2012). The patient-specific functional scale: psychometrics, clinimetrics, and application as a clinical outcome measure. J Orthop Sports Phys Ther.

[38] Selby D, Cascella A, Gardiner K, Do R, Moravan V, Myers J (2010). A single set of numerical cutpoints to define moderate and severe symptoms for the Edmonton Symptom Assessment System. J Pain Symptom Manag.

[39] Zwarenstein M, Treweek S, Gagnier JJ, Altman DG, Tunis S, Haynes B (2008). Improving the reporting of pragmatic trials: an extension of the CONSORT statement. BMJ..

[40] Nagakumar P, Doull I (2012). Current therapy for bronchiolitis. Arch Dis Child.

[41] Sveriges riksdag. Lag (2003:460) om etikprövning av forskning som avser människor Stockholm: The Swedish parliament (Riksdag); 2003 [cited 2019 28 August]. Summary in English p 31-44. Available from: https://www.riksdagen.se/sv/dokument-lagar/dokument/svensk-forfattningssamling/lag-2003460-om-etikprovning-av-forskning-som_sfs-2003-460.

