# Supplementary material for: The effect of physiotherapy including frequent changes of body position and stimulation to physical activity for infants hospitalised with acute airway infections. Study protocol for a randomised controlled trial
Source: Trials. 2020 Sep 21;21:803. doi: 10.1186/s13063-020-04681-9 (PMC7504844; doi:10.1186/s13063-020-04681-9)
Supplement: Supplementary file 2 — Additional file 2. [file 13063_2020_4681_MOESM2_ESM.docx]

**Observationsprotokoll andningsstudien.**

**Grupp Fysioterapi dag 1.**

| Patientens namn: |
| --- |
| Personnummer: |

| Dagens datum: |
| --- |

Daglig vikt (kl…….):__________________ Dagligt vätskebehov:______________________

Har **inhalerat:** Ja / Nej (ringa in det som gäller för dygnet)

Barnet är **aktivt** (ex kryper, sätter sig/ställer sig, går)? Ja / Nej (ringa in det som gäller)

Vätskelista:

| Klockan: |  |  |  |  |  |  |  |  |  |  |  |  |  |
| --- | --- | --- | --- | --- | --- | --- | --- | --- | --- | --- | --- | --- | --- |
| Amning/flaska (g/ml) |  |  |  |  |  |  |  |  |  |  |  |  |  |
| Sond (ml) |  |  |  |  |  |  |  |  |  |  |  |  |  |

**Behandling** med fysioterapeut/sjukgymnast 20 minuter

| **Tidpunkt/  klockan:**  2: a obs efter 20 min sedan var 3:e (2:a) timme | 1:a obs | 2:a obs |  |  |  |  |  |  |  |  |  |  |  |
| --- | --- | --- | --- | --- | --- | --- | --- | --- | --- | --- | --- | --- | --- |
| Saturation i upprätt ställning vid 2:a obs |  |  |  |  |  |  |  |  |  |  |  |  |  |
| Puls |  |  |  |  |  |  |  |  |  |  |  |  |  |
| Andn frekvens |  |  |  |  |  |  |  |  |  |  |  |  |  |
| Tillsatt syrgas (**%** eller **l/min**) |  |  |  |  |  |  |  |  |  |  |  |  |  |
| Högflöde i näsgrimma (l/min) |  |  |  |  |  |  |  |  |  |  |  |  |  |
| Väsande ljud (0-3)  **0**:Inga  **1**: Slut-utandning  **2**: Hela utandning  **3**: In och utandning |  |  |  |  |  |  |  |  |  |  |  |  |  |
| Allmäntillstånd (0-3  **0**: Vanligt  **3**: Irriterad/slö/nedsatt matintag |  |  |  |  |  |  |  |  |  |  |  |  |  |
| Indragningar (**0**=inga/ **B**uk/**H**als) |  |  |  |  |  |  |  |  |  |  |  |  |  |
| Näsvingespel **J**a**/N**ej |  |  |  |  |  |  |  |  |  |  |  |  |  |
| Föräldraskattning (**0-10**) mående ej på natten |  |  |  |  |  |  |  |  |  |  |  |  |  |
| Föräldr skattn mat **0-3** |  |  |  |  |  |  |  |  |  |  |  |  |  |

Föräldrars skattning: Var god vänd på pappret för skalorna!

Utskrivning till: hemmet eller intensivvårdsavdelning (ringa in aktuellt), klockan______

Förälders skattning: **Mående** (peka på siffra)**:**

I____I_____I_____I_____I_____I_____I_____I_____I_____I_____I

0 1 2 3 4 5 6 7 8 9 10

Som vanligt Mycket
 påverkad/sjuk

Förälders skattning: **Mat** (ange hur ditt barn äter)

**0** = äter som vanligt

**1** = äter mindre än vanligt

**2** = äter ingenting själv

**Kontaktperson för forskningsstudien:**

Sonja Andersson Marforio, leg sjukgymnast, doktorand Tel: 076-8092399
mail: [sonja.andersson_marforio@med.lu.se](mailto:sonja.andersson_marforio@med.lu.se)
