# Supplementary material for: The effect of physiotherapy including frequent changes of body position and stimulation to physical activity for infants hospitalised with acute airway infections. Study protocol for a randomised controlled trial
Source: Trials. 2020 Sep 21;21:803. doi: 10.1186/s13063-020-04681-9 (PMC7504844; doi:10.1186/s13063-020-04681-9)
Supplement: Supplementary file 3 — Additional file 3. [file 13063_2020_4681_MOESM3_ESM.pdf]

Skånes universitetssjukhus
En del av Region Skåne

Physiotherapist
Sonja Andersson Marforio

20/11/2017

BREATHING TREATMENT FOR SMALL CHILDREN

- **Inhalation,** always with the child in a sitting position or as upright as possible. The child must not have a comforter in their mouth and the mask should be tight against the face!

- Change the position/posture regularly. The lungs are aired differently depending on the position of the body.

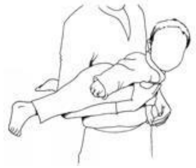

- Rock the child on a large ball or on the knee in different positions (lying on their side, stomach, sitting). Press lightly on the child's chest to give extra stimulation when exhaling.

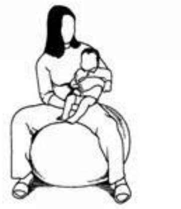 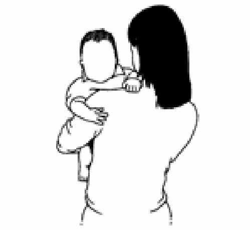 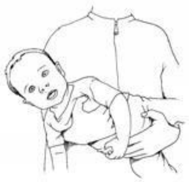

- Help/entice the child to move their arms and legs. Leg activity and arm movements above the head stimulate deep breathing.

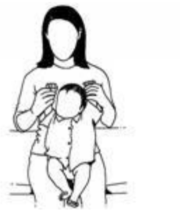 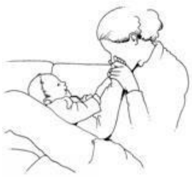

- Give the child support to cough by providing support over the child's stomach or by taking the child in your arms and "hugging".

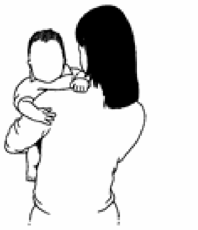

Repeat frequently, at least every other hour when the child is awake.
