# Supplementary material for: The effect of physiotherapy including frequent changes of body position and stimulation to physical activity for infants hospitalised with acute airway infections. Study protocol for a randomised controlled trial
Source: Trials. 2020 Sep 21;21:803. doi: 10.1186/s13063-020-04681-9 (PMC7504844; doi:10.1186/s13063-020-04681-9)
Supplement: Supplementary file 4 — Additional file 4. [file 13063_2020_4681_MOESM4_ESM.pdf]

Skånes universitetssjukhus
En del av Region Skåne

12/10/2018

Physiotherapist
Sonja Andersson Marforio
Children's Healthcare,
SUS

POSITION CHANGES FOR SMALL CHILDREN - PARENT INFORMATION

This is how you can help the child to take deep breaths and cough up phlegm.

- Give the child support to cough by placing the child over your shoulder and "hugging".

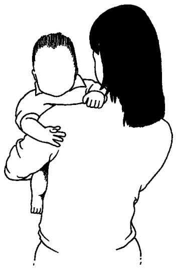

- Change the position/posture regularly. The lungs are aired differently depending on the position of the body. By all means let the child lie on their sides in bed too.

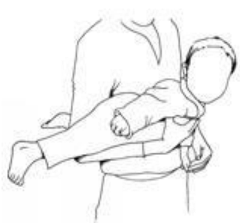

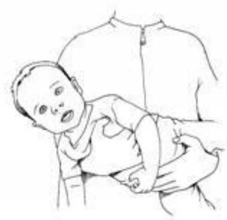

- Help the child to move their arms and legs. Leg activity and arm movements above the head stimulate deep breathing.
  You can stimulate the child to breathe deeply and to spontaneously cough through increased activity, for example, make mischief, tickling, singing.

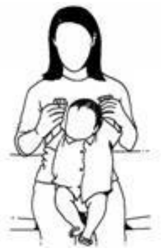

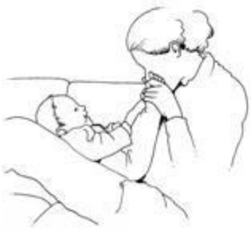

- Rock the child on your knee or up in your arms.

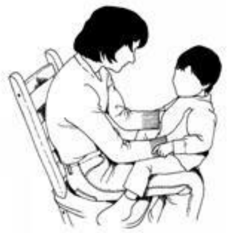

Repeat frequently, at least every other hour when the child is awake.
