# Supplementary material for: The effect of physiotherapy including frequent changes of body position and stimulation to physical activity for infants hospitalised with acute airway infections. Study protocol for a randomised controlled trial
Source: Trials. 2020 Sep 21;21:803. doi: 10.1186/s13063-020-04681-9 (PMC7504844; doi:10.1186/s13063-020-04681-9)
Supplement: Supplementary file 5 — Additional file 5. [file 13063_2020_4681_MOESM5_ESM.pdf]

Lund University
Department of Health Sciences
Physiotherapy

Lund University
Faculty of Medicine

Research study on physical therapy for infants.

Children who are in a hospital with respiratory distress often have difficulty breathing, have thick mucus, and may find it hard to eat normally. Sometimes physical therapy is used to treat these children, but it is not entirely known which methods help the children's condition. We are doing a study to evaluate physical therapy for infants who have difficulty breathing and are cared for at Skåne University Hospital and.

All children under 2 years of age who are admitted to the hospital for respiratory distress and who have previously been essentially healthy are asked to participate. If you choose to participate in the study, your child will be assigned to a control group that is treated as usual or to a group that receives additional care. The additional measures consist of changing the body positions and give some compressions of the chest, and will be performed by a physiotherapist or by the parents after they have practised it. Further action, in the form of inhalations or stimulation of deep breathing, will be used when needed. The measures will be performed every other hour during the day and once every night during the child's care period at the hospital. No additional blood tests or painful actions will be performed on the children.

Personal data will be handled only by the lead researcher, to ensure that all data are recorded correctly. Region Skåne is responsible for the child's personal data. The personal (social security) number will be saved together with information on how the child is at different times during the data collection period. The data will then be de-identified when entered into the statistics program. The results will be reported so that no individual person will be able to be identified. The results of the study are planned to be published in scientific journals. No compensation is paid for participation in the study. Ordinary patient injury insurance applies as with all health care in the hospital. The head of the research project is Region Skåne. According to PUL (Personal Data Act 1998: 204), the principal investigator must ensure that no unauthorised person can access the questionnaire or other personal data. According to PUL §26, the participant is entitled to apply for information from personal data processing, and this is done by writing to the personal information representative, Region Skåne, 291 89 Kristianstad. Such an application must be self-signed. You also have the right to get any incorrect personal information corrected. All information gathered in the study is handled in accordance with current confidentiality rules.

Participation in the research project is voluntary and you can withdraw at any time without special explanation. The child's care in general will not be affected, but will receive the usual care and checks. You may contact the researcher below to withdraw your consent to participate in the study.

If you would like more information about the study you are welcome to contact:

Sonja Andersson Marforio
Doctoral student

Eva Ekvall Hansson
Principal supervisor
Physiotherapist

Lund University
Department of Health Sciences/
Physiotherapy

Tel: 046-2221894

sonja.andersson_marforio@med.lu.se

Lund University
Department of Health Sciences/
Physiotherapy

Tel: 046-2221986

eva.ekvall-hansson@med.lu.se

Consent form for research study on physical therapy for infants.

I have been informed about the study and have had an opportunity to ask questions.

I hereby agree that my child will participate in the physical therapy study regarding breathing difficulties, at Skåne University Hospital. I am aware that participation in the study is completely voluntary and that I can, at any time, and without explanation, stop my participation without my child's care being affected in any way.

____

Date signature of parent/guardian 1

____

Date signature of parent/guardian 2

____

Date/Datum signature of responsible health care staff/vårdpersonal

Keep this part as your own copy!
